# 411. Phase 1/2 Trial of an Investigational Herpes Zoster Vaccine with CpG 1018® Adjuvant and Shingrix in Healthy Adults 50 to 69 Years of Age

**DOI:** 10.1093/ofid/ofaf695.018

**Published:** 2026-01-11

**Authors:** Oliver Medzihradsky, Rachel Graham, Paul Nguyen, Deon Smith, Paul Bird, John D Campbell, Mohamed El Idrissi, Ade Odueyungbo, Ouzama Henry, Robert Janssen

**Affiliations:** Dynavax Technologies, Emeryville, California; Innovate Clinical Research, Waitara, New South Wales, Australia; Emeritus Research Melbourne, Camberwell, Victoria, Australia; Canopy Clinical Research Northern Beaches, Brookvale, New South Wales, Australia; Emeritus Research Sydney, Botany, New South Wales, Australia; Dynavax Technologies, Emeryville, California; Dynavax Technologies, Emeryville, California; Dynavax Technologies, Emeryville, California; Dynavax Technologies, Emeryville, California; Dynavax Technologies Corporation, Emeryville, California

## Abstract

**Background:**

Herpes zoster (shingles) affects older adults due to waning cellular immunity. Z-1018, a CpG 1018^®^–adjuvanted gE subunit vaccine, is being developed to elicit efficacy comparable to Shingrix while offering improved tolerability. In a prior phase 1 trial, a CpG 1018^®^-adjuvanted vaccine with fixed-dosage commercial gE showed lower reactogenicity and comparable vaccine response rates (VRRs) to Shingrix.

**Table 1:**
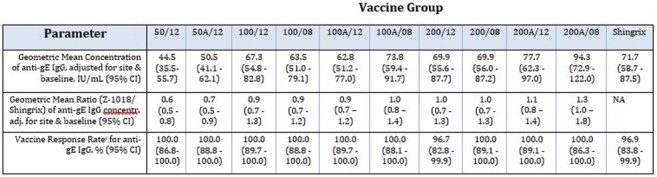
Anti-gE IgG Response at 4 Weeks Post Dose 2 (Per-Protocol Immunogenicity Analysis Population) Participants who received 2 vaccine doses, had no major protocol deviations, and had CD4+ T-cell assessment within the protocol-defined window. Groups (all Z-1018 groups used 6000 µg CpG 1018®/dose): 50/12: 50 mcg gE (0, 12 weeks); 50A/12: 50 mcg gE + Alum (0, 12 weeks); 100/12: 100 mcg gE (0, 12 weeks); 100/08: 100 mcg gE (0, 8 weeks); 100A/12: 100 mcg gE + Alum (0, 12 weeks); 100A/08: 100 mcg gE + Alum (0, 8 weeks); 200/12: 200 mcg gE (0, 12 weeks); 200/08: 200 mcg gE (0, 8 weeks); 200A/12: 200 mcg gE + Alum (0, 12 weeks); 200A/08: 200 mcg gE + Alum (0, 8 weeks); Shingrix (0, 8 and 0, 12 weeks). VRR=% participants with ≥4-fold increase in anti-gE IgG concentration from pre-vaccination baseline

**Methods:**

In an ongoing, randomized, observer-blinded phase 1/2 trial, adults ages 50–69 received 2 doses of Z-1018 (50, 100, or 200 µg Dynavax-manufactured gE adjuvanted with 6000 µg CpG 1018^®^ with/without 750 µg alum) given at an 8- or 12-week interval or Shingrix (10:1) at the same interval. Adverse events, post-injection reactions (PIRs) within 7 days, and immunogenicity (anti-gE IgG and activated gE-specific CD4⁺ T-cell responses) at 4 weeks after the second dose were evaluated. VRRs were defined as participants (%) with ≥4-fold increase in anti-gE IgG concentration over baseline and, separately, ≥2-fold increase in CD4⁺ T-cell frequency over baseline.

**Results:**

Among 441 participants (median age 58.0 years; 68.3% female), Z-1018 groups had lower rates of moderate-or-severe PIRs than Shingrix (local: 7.7–35.0% vs 52.6% for Shingrix; systemic: 17.5–46.2% vs 63.2% for Shingrix). No safety concerns were observed or reported. Anti-gE IgG VRRs (96.7–100%), and GMCs observed for the 100 µg and 200 µg gE formulations, were comparable to, and in some cases exceeded, those for Shingrix (Table 1). CD4^+^ data will be available for presentation at IDWeek.

**Conclusion:**

Z-1018 adjuvanted with 6000 µg CpG 1018^®^ +/- alum showed favorable tolerability and strong anti-gE IgG responses across a range of gE dosage levels and dosing intervals. These interim data support continued clinical development of Z-1018. Final trial results will be reported at a later date.

**Disclosures:**

All Authors: No reported disclosures

